# Prevalence of Health Effects Due to Disinfectant Exposure and Its Impact on Selected Physiological Parameters Among Class D Workers: A Descriptive Cross-Sectional Study

**DOI:** 10.7759/cureus.79994

**Published:** 2025-03-03

**Authors:** Vijaya R Kumbhar, Seema B Geddugol

**Affiliations:** 1 Obstetrics and Gynecology, Bharati Vidyapeeth (Deemed to be University) College of Nursing, Pune, IND

**Keywords:** class d workers, disinfectant exposure, health effects, physiological parameters, prevalence

## Abstract

Background: Cleaning and disinfection, particularly to reduce the risk of infection, constitute a significant portion of the work performed by medical personnel and other staff in hospital environments. In addition to the routine housekeeping tasks carried out by cleaning staff, these duties may involve medical specialists such as radiographers, respiratory therapists, physiotherapists, nurses, and class D workers responsible for cleaning and disinfecting surfaces and equipment. Various chemicals can be used for these tasks, including hydrogen peroxide, bleach, chlorine, and bacillocid. This study focused on estimating the prevalence of health effects of disinfectant exposure on physiological parameters among class D workers in selected hospitals in Sangli, Maharashtra.

Materials and methods: An analytical cross-sectional study was conducted using a quantitative descriptive approach between April 2024 and July 2024. A total of 270 participants were selected through a non-probability purposive sampling technique from the chosen hospitals, ensuring adherence to all ethical considerations. Data were collected using a disinfectant health effects on physiological parameters assessment checklist and a demographic information sheet for participants. After providing comprehensive details about the study, informed consent was obtained from all participants. The analysis was conducted using frequency and percentage distributions.

Results: Out of 270 class D workers, the survey found that 45.56% (123) of the participants reported having a sore nose due to hydrogen peroxide disinfectant exposure, 42.59% (115) reported throat irritation from glutaraldehyde disinfectant exposure, 40% (108) reported a persistent cough from sodium hypochlorite exposure, 37.4% (101) reported headaches from isopropyl alcohol disinfectant exposure, and 45.92% (124) reported eye irritation from benzalkonium chloride disinfectant exposure. Additionally, 59.62% (161) experienced skin irritation due to exposure to orthophthalaldehyde, and 58.15% (157) experienced nasal issues due to the bacillocid solution. Furthermore, 29.26% (79) experienced throat irritation from exposure to hypochlorite disinfectants, 44.05% (119) experienced nausea due to choline exposure, and 50.74% (137) experienced nausea from bleach solution exposure for more than six hours during their previous five years of employment.

Conclusions: The study concluded that most class D workers in Sangli hospitals experienced physical health effects, including irritation of the throat, nose, upper respiratory tract, eyes, and skin, as well as nausea. Identifying optimal practices to minimize worker exposure while ensuring patient safety is crucial. Based on the present study’s findings, hospital managers should develop safety resources, conduct educational campaigns, and provide class D employees with the necessary training to manage disinfectant exposure effectively.

## Introduction

Cleaning is an occupational risk factor for asthma among healthcare professionals, according to several studies. Several chemicals found in cleaning and disinfection products, including quaternary ammonium compounds, ethanolamine, chlorhexidine, glutaraldehyde, orthophthalaldehyde (OPA), hexachlorophene, and chloramine-T, can cause or exacerbate asthma due to their sensitizing or irritating properties [1].

Reactive oxygen species and formaldehyde are oxidation products that may worsen health effects when cleaning agents interact with common ozone [2].

Spray cleaning and disinfection solutions typically contain complex chemical mixtures, including volatile organic compounds, primarily used as solvents and fragrances, preservatives, and disinfectants. Consequently, inhalation exposure is likely higher after spray application than other liquid application methods [3].

Healthcare workers are exposed to high concentrations of various cleaning and disinfection chemicals. Hospitals are increasing disinfection efforts to protect patients from healthcare-associated infections (HAIs). Growing evidence shows that exposure to cleaning products and disinfectants raises the risk of respiratory diseases, including asthma. Although the exact causes remain unclear, bleach, quaternary ammonium compounds (quats), ammonia, medical equipment cleaning supplies, and spray-form products have all been linked to a higher risk of respiratory issues [4].

The health and safety of personnel should be considered when selecting cleaning and disinfection supplies. Disinfection chemicals help reduce healthcare-acquired infections in healthcare settings. A request was made to conduct a health hazard evaluation at a hospital in Pennsylvania regarding the use of a novel surface cleaning solution containing hydrogen peroxide, acetic acid, and peroxyacetic acid. Concerns were raised about hospital environmental services staff being exposed to the cleaning solution, with reported symptoms including skin burns, headaches, coughing, burning eyes, nose and throat irritation, and asthma flare-ups [5].

The most commonly reported health outcomes were shortness of breath (16%), watery eyes (46%), nasal issues (41%), asthma-like symptoms (28%), and the use of allergy medication (16%). Thirty workers (44%) reported experiencing at least one work-related health problem, most commonly watery eyes (29%) or nasal issues (22%). Among the ten respondents who self-reported a doctor's diagnosis of asthma, six stated that something at work aggravated or triggered their asthma, with three specifically identifying the disinfection product as the cause [6].

From the onset of the outbreak, preventing the spread of COVID-19 was a top priority, necessitating the use of health measures such as sanitizers and disinfectants. While disinfectants effectively prevent and manage COVID-19, significant concerns remain regarding their potential impact on human health [7].

Aldehydes, hydrogen peroxide, quaternary ammonium compounds, phenol-based disinfectants, oxidizing agents, detergents, chlorine-releasing agents (such as sodium hypochlorite and per-chlorine), and alcohol-based materials are commonly used. However, most sanitizers and disinfectants can pose health risks to humans, as they contain oxidizing agents, quaternary ammonium cations, and chlorine-releasing agents, among other toxic and corrosive compounds [8].

In recent years, cleaning has been recognized as an occupational hazard, as cleaning workers are more likely to experience respiratory issues, including asthma and asthma-like symptoms [9].

Therefore, the present study aims to conduct a comprehensive survey to assess the extent of health effects on various physiological parameters caused by disinfectant exposure, along with the demographic characteristics of Class D workers in selected hospitals in Sangli.

## Materials and methods

This study, conducted in a hospital, aimed to determine the frequency of health impacts on specific physiological parameters resulting from disinfectant exposure. A quantitative descriptive research approach was used. Class D workers were selected based on specific criteria, including having worked for at least one year, being able to read and write Marathi, being either male or female, and being willing to provide written informed consent. The study excluded class D workers with a history of severe comorbidities and those receiving occupational safety training. A total of 270 participants were recruited, with an anticipated sample size of 246, accounting for a 10% dropout or attrition rate. Participants were selected using a non-probability purposive sampling technique. Univariate analysis was conducted.

The data collection tools were divided into two sections. The first section, which included seven questions, focused on the demographic characteristics of class D workers, such as age, gender, work experience, frequency of daily exposure to disinfectants, duration of exposure, reason for disinfectant use, and use of personal protective equipment (PPE). The second section included the disinfectant exposure health effects evaluation checklist, which was used to gather data on physiological indicators. Data were collected on disinfectants such as hydrogen peroxide, glutaraldehyde, sodium hypochlorite, isopropyl alcohol, benzalkonium chloride, OPA, bacillocid, choline, and bleach. The test-retest methodology was used to assess the reliability of the disinfectant exposure health effects evaluation checklist, yielding a high reliability score (r=0.86). The scores were interpreted based on the frequency and percentage of health effects on physiological parameters caused by disinfectants. All data were collected between April and July 2024. Each selected physiological parameter was scored using a "yes" or "no" response.

The study followed a survey design (non-interventional), which is not considered a clinical trial and, therefore, could be waived. However, the study title was approved by the Institutional Ethics Committee of Bharati Vidyapeeth (Deemed to be University), College of Nursing, Sangli, which is registered with the Department of Health Research, New Delhi (Reg. No. EC/NEW/INST/2024/MH/0444be). All study participants were duly informed about the purpose of the study, and written informed consent was obtained from each subject and the hospital authorities. Confidentiality was maintained throughout the study, and participants and hospital authorities were assured of data confidentiality and the anonymity of their organization.

## Results

The study's findings are arranged and explained in three sections: demographic data, the frequency and percentage of disinfectant-induced health effects on physiological parameters, and a comparison of the disinfectant exposure effect based on specific physiological parameters among most class D workers. For data analysis of the study, all the gathered data were coded and computed in Microsoft Excel (Microsoft Corporation, Redmond, WA, USA). In the analysis tables, the researchers considered only the "yes" responses for the health effect assessment due to disinfectants, and the data was summarized using multivariate analysis in frequency and percentage distribution.

According to the findings related to the demographic variables, the univariate analysis method was used. The age distribution reveals that most participants (47.78%, 129) were between the ages of 30 and 40 years, followed by 45.19% (122) between 40 and 50 years, and 7.03% (19) between 50 and 60 years. The gender-wise distribution indicated that most participants (50.74%, 137) were female, while 49.29% (133) were male. Regarding work experience, 47.78% (129) of participants had more than five years of experience, 43.33% (117) had up to four years, and only 5.19% (14) had between two and four years of experience. The data revealed that all participants (100%, 270) were exposed to disinfectants daily and faced workplace hazards. Meanwhile, 56.67% (153) were exposed for less than six hours per day, while 43.34% (117) were exposed for more than six hours per day. Regarding the frequency of disinfectant use, 59.62% (161) reported cleaning surfaces three times per day, 46.92% (125) reported cleaning medical instruments four times per day, and 24.07% (65) were exposed to fumigation of the wards and operating theaters daily. Of the 270 participants, 70.74% (191) did not use PPE kits regularly, while only 29.25% (79) used gloves and masks. All the findings are presented in Table 1.

**Table 1:** Multivariate analysis of the demographic characteristics by frequency and percentage of class D workers (n=270) OT: operation theaters, PPE: personal protective equipment

Sr. No.	Demographic characteristics of the participants	N	Percentage (%)
1	Age in years		
1.1	30-40	129	47.78
1.2	40-50	122	45.19
1.3	50-60	19	7.03
2	Gender		
2.1	Male	133	49.26
2.2	Female	137	50.74
3	Working experience in years		
3.1	1-2	10	3.7
3.2	2-4	14	5.19
3.3	4-5	117	43.33
3.4	More than 5 years	129	47.78
4	No. of times exposed to disinfectants per day (3 times/day)	270	100
5	Exposed to any disinfectant hazards		
5.1	Yes	270	100
5.2	No	0	0
6	Duration of exposure		
6.1	< 6 hours	153	56.67
6.2	> 6 hours	117	43.34
7	Usage of disinfectants		
7.1	Cleaning surfaces (3 times/day)	161	59.62
7.2	Cleaning medical instruments (4 times/day)	125	46.92
7.3	Fumigation of wards, OT, etc.	65	24.07
8	Using a PPE kit while handling disinfectants		
8.1	Yes	79	29.25
8.2	No	191	70.74

The analysis shows that among the 270 class D workers, the majority (45.56%, 123) reported having a sore nose. In contrast, 38.88% (105) reported having a sore throat, 5.56% (15) reported feeling nauseous, 5.18% (14) reported feeling sick, and 4.45% (12) reported vomiting. Additionally, 15.18% (41) of the participants experienced a cough after being exposed to hydrogen peroxide disinfectants for more than six hours over the past five years of employment. Regarding exposure to glutaraldehyde disinfectants, most (42.59%, 115) reported throat irritation, while 27.40% (74) experienced nose irritation. Additionally, 12.22% (33) reported difficulty breathing, 10.37% (28) experienced sneezing, 6.48% (17) had eye itching, 5.55% (15) suffered from asthma attacks, and 4.8% (13) reported a burning sensation in the eyes. A small percentage (4.49%, 11) reported a skin rash. Furthermore, 40% (108) reported having a persistent cough when surveyed for sodium hypochlorite disinfectant exposure. In comparison, 22.9% (62) had difficulty breathing, 21.85% (59) experienced constant headaches, and 15.55% (42) reported skin itching when the solution came into contact with their skin. A small percentage (7.7%, 21) reported inhalation-related stomach irritation. Finally, findings on isopropyl alcohol disinfectant exposure showed that most (37.4%, 101) reported headaches. Additionally, 20% (54) experienced nose irritation, 14.07% (38) had throat irritation, 11.48% (31) reported confusion, 10.74% (29) felt dizzy while using the solution, 10% (27) developed skin rashes, and 6.77% (21) reported a loss of coordination. All these findings are summarized in Table 2.

**Table 2:** Multivariate analysis of the health effects of disinfectant solutions (hydrogen peroxide, glutaraldehyde, sodium hypochlorite, and isopropyl alcohol) on selected physiological parameters in terms of frequency and percentage (n=270)

Sr. No.	Disinfectants and their effect on physiological parameters	N	Percentage (%)
1	Hydrogen peroxide		
1.1	Irritation of throat	105	38.88
1.2	Irritation of nose	123	45.56
1.3	Cough	41	15.18
1.4	Breathing difficulty	12	4.45
1.5	Nausea	15	5.56
1.6	Vomiting	14	5.18
2	Glutaraldehyde		
2.1	Irritation of throat	115	42.59
2.2	Nose Irritation	74	27.40
2.3	Asthma	15	5.55
2.4	Breathing difficulty	33	12.22
2.5	Sneezing	28	10.37
2.6	Burning eyes	13	4.8
2.7	Itching of eyes	17	6.48
2.8	Rash on skin	11	4.49
3	Sodium hypochlorite		
3.1	Breathing difficulty	62	22.9
3.2	Cough	108	40
3.3	Headache	59	21.85
3.4	Itching of skin	42	15.55
3.5	Irritation of stomach	21	7.7
4	Isopropyl alcohol		
4.1	Irritation of throat	38	14.07
4.2	Irritation of nose	54	20
4.3	Headache	101	37.4
4.4	Skin rash	27	10
4.6	Dizziness	29	10.74
4.7	Loss of coordination	21	7.77
4.8	Confusion	31	11.48

Similarly, among the 270 class D workers, 45.92% (124) reported eye irritation, 24% (65) reported stomach irritation, 17.48% (47) reported skin irritation, 4.44% (12) reported difficulty breathing, 4.44% (12) reported wheezing from fumes, and 4.08% (11) reported nasal irritation, swelling, and breathing difficulties as a result of exposure to benzalkonium chloride disinfectants. Additionally, the majority (59.62%, 161) experienced skin irritation, 15.56% (42) reported eye discomfort, and 14.44% (39) had nasal irritation. About 10.38% (28) of participants reported having asthma due to exposure to OPA. Furthermore, the majority (58.15%, 157) experienced nasal issues, 28.15% (76) reported throat irritation, and 13.7% (37) complained of coughing while exposed to the bacillocid solution. All these findings are presented in Table 3.

**Table 3:** Multivariate analysis of the health effects of disinfectant solutions (benzalkonium chloride, OPA, and bacillocid) on selected physiological parameters in terms of frequency and percentage (n=270) OPA: orthophthalaldehyde

Sr. No.	Disinfectants and their effect on physiological parameters	N	Percentage (%)
1	Benzalkonium chloride		
1.1	Irritation and swelling inside the nose	11	4.08
1.2	Wheezing	12	4.44
1.3	Breathing difficulty	11	4.08
1.4	Skin irritation	47	17.48
1.5	Eyes irritation	124	45.92
1.6	Stomach irritation	65	24
2	OPA		
2.1	Irritation of nose	39	14.44
2.2	Asthma	28	10.38
2.3	Skin irritation	161	59.62
2.4	Eyes irritation	42	15.56
3	Bacillocid		
3.1	Irritation of the throat	76	28.15
3.2	Irritation of the nose	157	58.15
3.3	Cough	37	13.7

**Figure 1:**
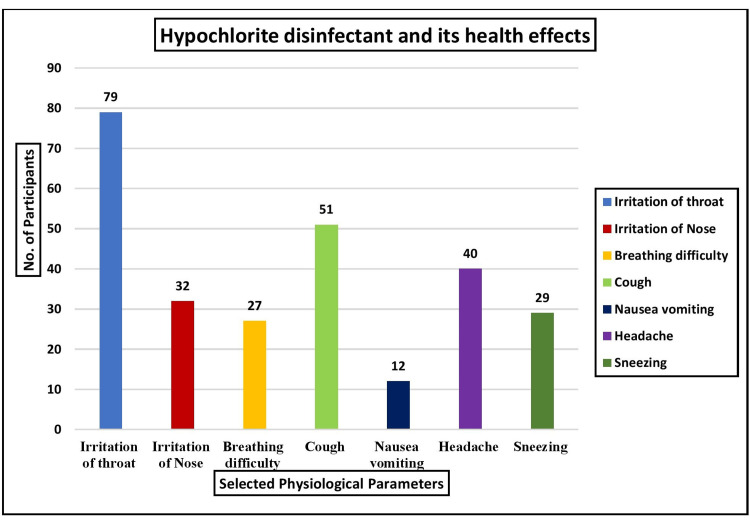
Multivariate analysis of the health effects of hypochlorite on selected physiological parameters in terms of frequency (n=270)

According to the study analysis, most (29.26%, 79) reported throat irritation. Additionally, 18.89% (51) experienced coughing, 14.81% (40) had headaches, 11.86% (32) reported nasal irritation, 10.74% (29) experienced sneezing, 10% (27) had difficulty breathing, and 4.44% (12) reported nausea and vomiting due to exposure to hypochlorite disinfectants. These findings are illustrated in Figure 1.

**Figure 2:**
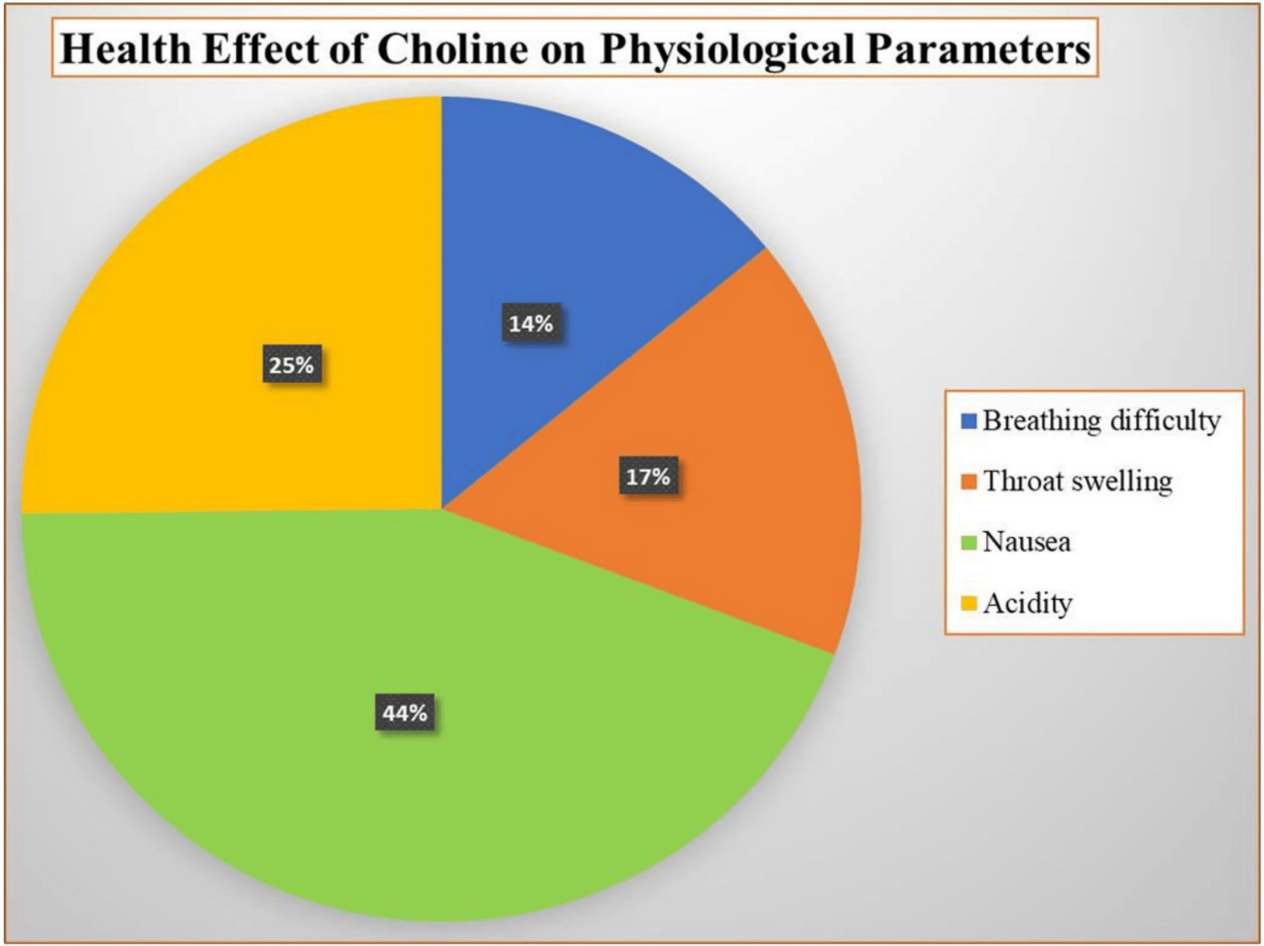
Multivariate analysis of the health effects of choline on selected physiological parameters as a percentage (n=270)

The majority of participants (44.05%, 119) reported complaints of nausea, while 25.19% (68) experienced acidity, and 16.67% (45) reported throat swelling due to exposure to the choline solution. All these findings are shown in Figure 2.

**Figure 3:**
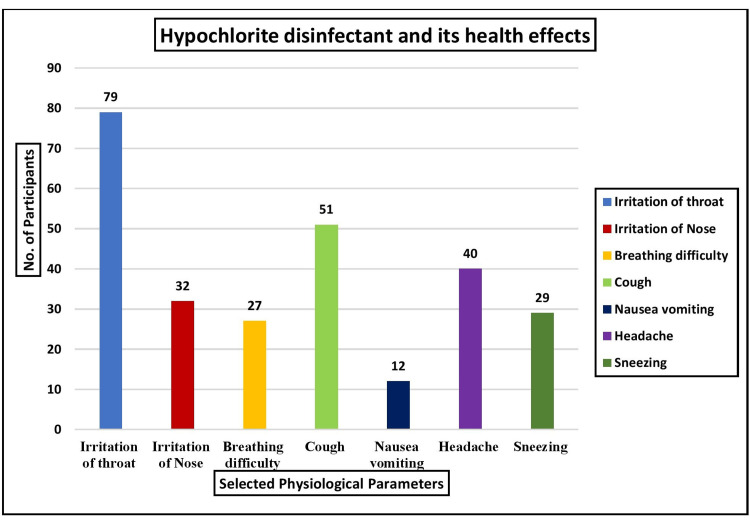
Multivariate analysis of the health effects of bleach on selected physiological parameters in terms of percentage (n=270)

Most participants (50.74%, 137) reported complaints of nausea. Additionally, 14.82% (40) had a cough, 13.7% (37) reported shortness of breath, and 10.74% (29) had health issues such as bronchitis. Furthermore, 10% (27) reported complaints of skin ulcers due to exposure to the bleach solution. All these findings are shown in Figure 3.

**Figure 4:**
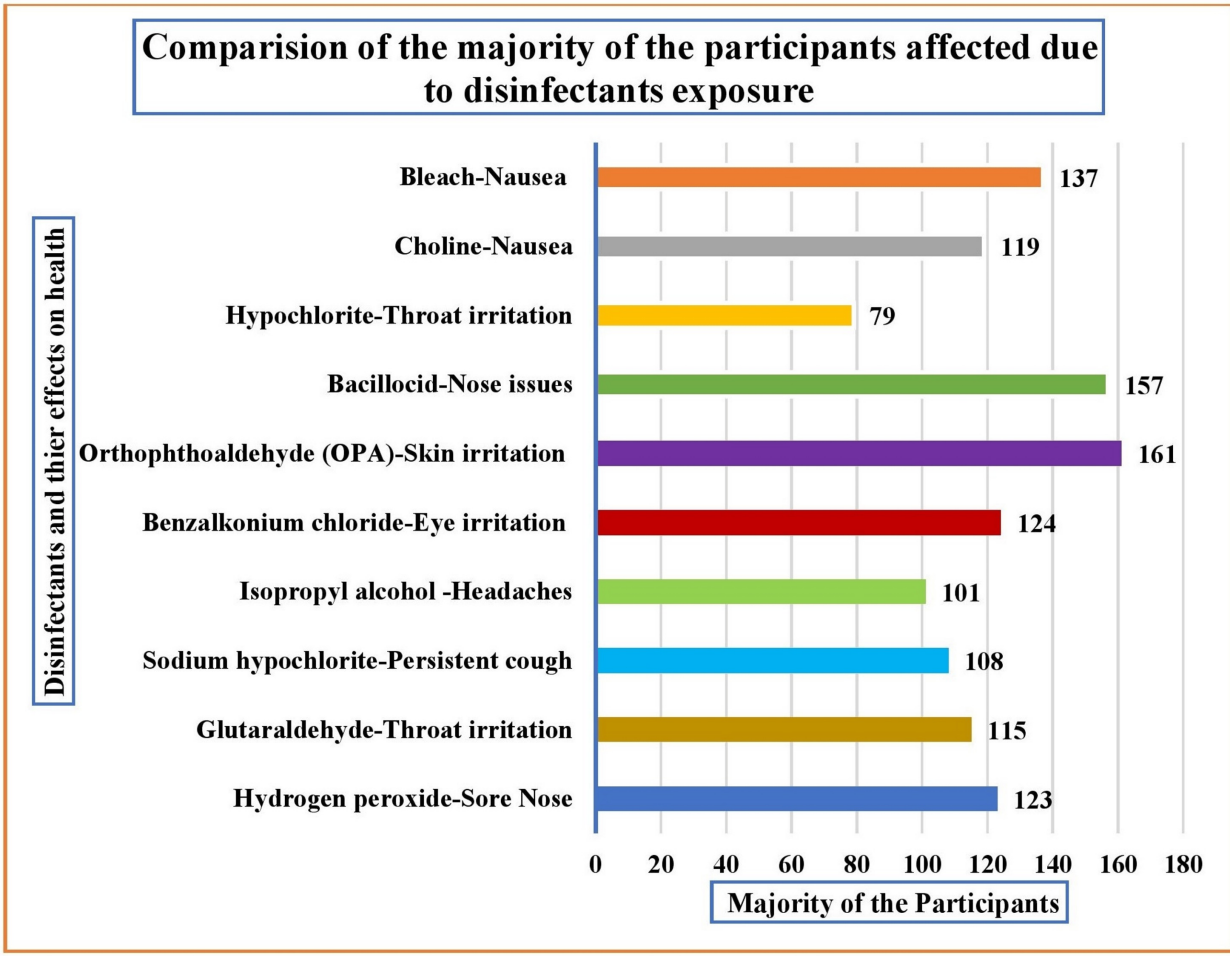
Multivariate analysis of the frequency comparisons among most participants affected by disinfectant exposure (n=270) OPA: orthophthalaldehyde

The majority reported disinfectant-induced health effects on selected physiological parameters. About 42.59% (115) reported throat irritation from glutaraldehyde disinfectant exposure, 40% (108) reported a persistent cough from sodium hypochlorite exposure, 37.4% (101) reported headaches from isopropyl alcohol disinfectant exposure, and 45.92% (124) reported eye irritation from benzalkonium chloride disinfectant exposure. Additionally, 59.62% (161) experienced skin irritation from exposure to OPA, while 58.15% (157) experienced nasal issues from exposure to the bacillocid solution. Furthermore, 29.26% (79) experienced throat irritation due to hypochlorite disinfectants, 44.05% (119) experienced nausea due to choline, and 50.74% (137) experienced nausea as a result of using a bleach solution. All these findings are presented in Figure 4.

## Discussion

The study findings aligned with the stated results that eight disinfectant exposures were included in the current analysis, namely alcohol, quaternary ammonium compounds, ammonia, formaldehyde, glutaraldehyde, bleach/chlorine, latex gloves, and sprays. Among the 327 former hospital employees surveyed, 176 were exposed, and their ratings were examined. Notably, for formaldehyde, ammonia, alcohol, and quaternary ammonium compounds (16.6% vs. 70.9%, p<0.0001), self-reported exposure was underestimated compared to expert evaluation. Occupational exposure to cleaning and disinfection agents in hospitals is frequent and significant. The study revealed a substantial underestimation of self-reported exposure and a lack of knowledge regarding product components. It concluded that proper attention should be given to the side effects induced by self-reported exposure, and an occupational hazard protocol should be developed to minimize these risks [1].

The findings also aligned with a study conducted on 8,851 nurses regarding the use of disinfectants, which was evaluated through a questionnaire. The American Medical Association database provided information on hospital characteristics. Hospital employment was associated with decreased spray use (0.74 (0.66-0.82)) but higher disinfectant use (OR: 2.06 (95% CI: 1.89-2.24)). Nurses employed in smaller hospitals (fewer than 50 beds) used disinfecting agents more frequently (1.69 (1.23-2.32)) than those in hospitals with more than 200 beds. Perhaps due to their shift duties, nurses in smaller hospitals were more likely to use disinfectants. Regional and hospital-size-specific differences in spray use should be a focus of future occupational asthma prevention initiatives [10].

The right-to-know hazardous substance fact sheet supports these study findings, revealing that hydrogen peroxide causes short-term health effects such as nasal and throat irritation due to inhalation, skin irritation, and burning sensation due to contact, coughing, and shortness of breath. Long-term exposure can lead to pulmonary edema, headaches, dizziness, nausea, and vomiting [11].

Other study findings discovered that participants exposed to disinfecting products had a higher prevalence of watery eyes and wheezing. Half of the employees reported that workplace conditions caused or exacerbated their nasal problems. A 2007 case study documented two endoscopy nurses exposed to a mixture of hydrogen peroxide and peracetic acid, resulting in coughing, wheezing, and shortness of breath [12].

The findings on hypochlorite use are similar. Due to its superior bleaching and sterilizing properties in residential and commercial settings, sodium hypochlorite is frequently used as a primary ingredient in cleaning products. In addition to bleaching, it is also utilized as a disinfecting agent during the COVID-19 pandemic, in wastewater treatment, and for food factory sterilization. Most case studies discuss the negative health effects of acute high-concentration exposure [13].

Similar findings were observed among workers exposed to infection control procedures requiring products containing hydrogen peroxide, peracetic acid, and acetic acid, which may cause adverse health effects. Environmental cleaning and disinfection is a crucial component of a comprehensive HAI prevention strategy, as HAIs pose a significant risk to patient safety. However, addressing worker concerns about occupational illness may allow healthcare facilities to create a safer environment for employees while also protecting patients from HAIs. This study emphasized that disinfectant exposure should be considered when assessing healthcare professionals for occupational illnesses. Further research is required to characterize product exposure, related health impacts, and control strategies [14].

Multiple studies support similar findings regarding workers exposed to cleaning or disinfecting solutions, showing higher asthma rates and asthma-like respiratory symptoms in various work environments. Hospital staff may be susceptible to respiratory and skin complaints due to their frequent cleaning and disinfection tasks. The findings suggest that hospital employees exposed to cleaning and disinfecting agents should follow preventive measures to lower their risk of developing asthma and similar symptoms [15].

We, as researchers, acknowledge the limited scope of our current investigation. Using a survey research design, the study findings were restricted to 270 participants from specific hospitals in Sangli. A larger sample size could have allowed for a comparison of class D employees from government and private hospitals, thereby increasing the generalizability of the results. Although the study focused on class D employees, other medical professionals might also experience similar exposures. Additionally, only a few physiological measures were available to the researcher. However, disinfectant-induced health impacts on particular systems can be further evaluated, and a protocol with therapeutic and intervention packages can be developed for all class D workers regularly exposed to disinfectants in various hospital units.

## Conclusions

There should be a protocol for workplace exposure that includes standard recommended workplace practices, such as tailored education on handling disinfectant hazards safely, assessment of airborne biochemical concentrations, provision of eye wash fountains and wash showers in case of skin contact, regular training on proper handwashing practices, changing into clean clothing in case of contamination with disinfectants, and ensuring workplace facilities for washing contaminated clothing instead of washing them at home. Additionally, employees should avoid eating and drinking in disinfectant storage areas and use appropriate PPE, including gloves, protective clothing, eye protection, and respiratory protectors. Furthermore, they should receive training from fire brigade agencies on handling fire incidents related to disinfectant exposure, as these substances can be explosive.

## Appendices

The information-gathering tool was divided into two sections. Section I focused on class D workers' demographic factors, including age, gender, work experience, number of times exposed to disinfectants per day, duration of exposure, reason for using disinfectants, and use of PPE kits. Participants were asked to place a (√) next to their selected options. The responses were calculated in terms of frequency and percentage. All demographic characteristics are presented in Table 4.

**Table 4:** Demographic characteristics of the participants

Sr. No.	Demographic variables	Response
1	Age in years	
1.1	30-40	
1.2	40-50	
1.3	50-60	
2	Gender	
2.1	Male	
2.2	Female	
3	Working experience in years	
3.1	1-2	
3.2	2-4	
3.3	4-5	
3.4	More than 5 years	
4	No. of times exposed to the disinfectants per day (3 times/day)	
5	Have you faced any disinfectant hazards?	
5.1	Yes	
5.2	No	
6	Duration of exposure	
6.1	< 6 hours	
6.2	> 6 hours	
7	Usage of disinfectants	
7.1	Cleaning surfaces (3 times/ day)	
7.2	Cleaning medical instruments (4 times/day)	
7.3	Fumigation of wards, OT, etc.	
8	Have you used a PPE kit while handling the disinfectants?	
8.1	Yes	
8.2	No	

Section II used the disinfectant exposure health effects evaluation checklist to gather data on physiological indicators. Data were collected on disinfectants such as hydrogen peroxide, glutaraldehyde, sodium hypochlorite, isopropyl alcohol, benzalkonium chloride, orthophthalaldehyde (OPA), bacillocid, choline, and bleach. The test-retest methodology was used to evaluate the reliability of the disinfectant exposure health effects evaluation checklist, and the results showed that it was highly reliable (r=0.86). The scores were interpreted based on the frequency and percentage of health effects on physiological parameters caused by disinfectants. Each physiological parameter was scored as "yes" or "no." The complete questionnaire is presented in Table 5, which displays the disinfectant exposure health effects evaluation checklist for selected physiological parameters.

**Table 5:** Questionnaire on disinfectants and their health effects: evaluation checklist for selected physiological parameters OPA: orthophthalaldehyde

Sr. No	Disinfectants and their health effects	Yes	No
		1	0
1	Hydrogen peroxide		
1.1	Irritation of throat		
1.2	Irritation of nose		
1.3	Cough		
1.4	Breathing difficulty		
1.5	Nausea		
1.6	Vomiting		
2	Glutaraldehyde		
2.1	Irritation of throat		
2.2	Nose irritation		
2.3	Asthma		
2.4	Breathing difficulty		
2.5	Sneezing		
2.6	Burning eyes		
2.7	Itching of eyes		
2.8	Rash on skin		
3.	Sodium hypochlorite		
3.1	Breathing difficulty		
3.2	Cough		
3.3	Headache		
3.4	Itching of skin		
3.5	Irritation of stomach		
4.	Isopropyl alcohol		
4.1	Irritation of throat		
4.2	Irritation of nose		
4.3	Headache		
4.4	Skin rash		
4.5	Headache		
4.6	Dizziness		
4.7	Loss of coordination		
4.8	Confusion		
5	Benzalkonium chloride		
5.1	Irritation and swelling inside the nose		
5.2	Wheezing		
5.3	Breathing difficulty		
5.4	Skin irritation		
5.5	Eyes irritation		
5.6	Stomach irritation		
6	OPA		
6.1	Irritation of nose		
6.2	Asthma		
6.3	Skin irritation		
6.4	Eyes irritation		
7	Bacillocid		
7.1	Irritation of the throat		
7.2	Irritation of the nose		
7.3	Cough		
8.	Hypochlorite		
8.1	Irritation of throat		
8.2	Irritation of nose		
8.3	Breathing difficulty		
8.4	Cough		
8.5	Nausea vomiting		
8.6	Headache		
8.7	Sneezing		
9.	Choline		
9.1	Breathing difficulty		
9.2	Thorat swelling		
9.3	Nausea		
9.4	Acidity		
10	Bleach		
10.1	Bronchitis		
10.2	Shortness of breath		
10.3	Nausea		
10.4	Skin ulcer		
10.5	Cough		
